# Translocating shRNA: a novel approach to RNA interference with Newcastle disease virus as viral vector

**DOI:** 10.1099/jgv.0.002127

**Published:** 2025-07-11

**Authors:** Wei Chin Koh, Khatijah Yusoff, Adelene Ai-Lian Song, Norazalina Saad, Tiong Kit Tan, Pheik-Sheen Cheow, Suet Lin Chia

**Affiliations:** 1 UPM-MAKNA Cancer Research Laboratory, Institute of Bioscience, Universiti Putra Malaysia, 43400 UPM Serdang, Selangor, Malaysia; 2 Department of Microbiology, Faculty of Biotechnology & Biomolecular Sciences, Universiti Putra Malaysia, 43400 UPM Serdang, Selangor, Malaysia; 3 Malaysia Genome & Vaccine Institute, National Institutes of Biotechnology Malaysia, Jalan Bangi, 43000 Kajang, Selangor, Malaysia; 4 MRC Translational Immune Discovery Unit, MRC Weatherall Institute of Molecular Medicine, University of Oxford, OX3 9DS, Oxford, UK; 5 Chinese Academy of Medical Sciences Oxford Institute, Nuffield Department of Medicine, University of Oxford, OX3 7BN, Oxford, UK

**Keywords:** Drosha, Newcastle disease virus (NDV), short hairpin RNA, viral vector, ZC3H12D protein

## Abstract

RNA interference is crucial in post-transcriptional gene silencing. Short hairpin RNA (shRNA) is particularly effective because it forms fully complementary matches with target mRNA, leading to its degradation. However, shRNA processing relies on nuclear microprocessors like Drosha, posing a challenge for RNA viral vectors that replicate exclusively in the cytoplasm. Although there have been reports of Drosha translocating to the cytoplasm upon viral infection, many RNA viruses, including Newcastle disease virus (NDV), remain inadequately studied in this context and, in some cases, fail to induce Drosha translocation for shRNA processing. In this study, we developed a novel approach to translocate an shRNA, expressed by NDV as an RNA viral vector, into the nucleus for Drosha processing. As a proof of concept, a recombinant NDV expressing the shRNA (rAF-shmcherry) with an AU-rich region at its 3′ end in the expression cassette was constructed. This shRNA targets a constitutively expressed mCherry gene in a colorectal cancer cell line, SW620-mC. We confirmed the presence of the AU-rich shRNA in the nuclei of the rAF-shmcherry-infected SW620-mC using reverse transcription PCR (RT-PCR). The gene-silencing effect of the shRNA was then evaluated at mRNA and protein levels, showing ~90% downregulation of the mCherry transgene at 24 h post-infection and 70% downregulation of mCherry protein in SW620-mC at 48 h post-infection. This study marks the first exploration of NDV as an shRNA viral vector, presenting a promising approach for shRNA translocation that could be applicable to various RNA viruses.

**Impact Statement**

We developed a novel method to facilitate Drosha processing by translocating short hairpin RNA (shRNA) transcripts, produced in the cytoplasm by recombinant Newcastle disease virus (NDV), into the nucleus. Our design incorporates an AU-rich region at the 3′ end of shRNA, promoting the binding of ZC3H12D protein for nuclear localization. This approach demonstrates NDV’s potential as a vector for shRNA delivery, enabling targeted mRNA silencing. Unlike prior studies that relied on Drosha translocation into the cytoplasm during RNA viral infection, our method is universally applicable to RNA viruses, expanding the viral vector option for RNA interference delivery. This study is the first to report the use of recombinant NDV as an shRNA delivery vector, independent of Drosha translocation.

## Data availability

The ZC3H12D expression in cells was obtained from the GeneCards bioinformatic database (https://www.genecards.org/cgi-bin/carddisp.pl?gene=ZC3H12D). The information about the subcellular location of ZC3H12D protein was retrieved from UniProtKB/Swiss-Prot (A2A288-ZC12D_HUMAN).

## Introduction

RNA interference (RNAi) is the biological process of mRNA degradation caused by complementary sequences, ds small interfering RNAs (siRNA) and knockdown of target gene expression. It plays a role in transcriptional and post-transcriptional gene regulation in eukaryotes and aids in the host antiviral response against RNA viruses [1]. In RNAi, there are several types of small RNAs, such as microRNA (miRNA), siRNA and short hairpin RNA (shRNA). They are similar to each other in terms of their function, which can cause targeted gene silencing through either translation inhibition or mRNA degradation, depending on the degree of its complementarity to the target gene [2]. Due to its efficient and extremely specific gene-silencing characteristics, RNAi has since become recognized as a straightforward, affordable and selective technique to regulate gene expression in human diseases.

A typical structure of shRNA consists of a paired antisense and sense strand joined by a loop of unpaired nucleotides. shRNA is a synthetic RNA molecule with a hairpin turn and possesses a strong affinity for its target [3]. The shRNA is transcribed by either the RNA polymerase II or III promoter [4,5] and transforms into siRNAs by utilizing the canonical miRNA biogenesis pathway [5]. Under RNA polymerase II, the primary transcript of shRNA (pri-shRNA) requires the microprocessor (Drosha/DGCR8) in the nucleus for processing into precursor shRNA (pre-shRNA). The pri-shRNA has a stem-loop structure that resembles a hairpin, where this structure is absent in siRNA. However, pre-shRNA is directly transcribed from RNA polymerase III, and the microprocessor processing is not necessary. Both pri-shRNA and pre-shRNA have a similar structure to pri- and pre-miRNA. Once produced, with the help of XPO5, the shRNA hairpin leaves the nucleus and is then processed by Dicer into ~22 bp siRNA duplexes by adhering to loop-counting, 5′-end and 3′-end rules, just as the pre-miRNA [5]. Finally, the guide strand of the siRNA, which is complementary to the target mRNA, loads onto the RNA-induced silencing complex (RISC), leading to gene silencing through mRNA degradation [6]. The silencing effect of shRNA or siRNA is usually better than that of miRNA, as they are purposefully designed to be fully complementary to the target mRNA, leading to mRNA degradation instead of translational inhibition, which has a weaker silencing effect [2].

Viral vectors, such as adenovirus, adeno-associated virus (AAV), retrovirus or lentivirus and baculovirus, have been reported to be used as the vectors carrying shRNA for gene silencing. By cloning an oligonucleotide containing the siRNA sequence into the viral or plasmid vectors, endogenous expression of shRNA can be achieved, which is then converted into siRNA in the cytoplasm [7]. Targeted therapies using shRNAs have been developed for many diseases, including human immunodeficiency virus type 1 infection, retinal abnormalities and different cancers [8,10]. Sliva and Schnierle [7] reported that in order to effectively transport shRNA, the delivery vehicle should enable endosomal/lysosomal escape and allow the payload to pass through the nuclear membrane [7]. However, the replication of most RNA viral genomes occurs in the cytoplasm, where the RNAs remain exclusively in the cytoplasm. This could be a challenge unless (i) the Drosha is translocated to the cytoplasm for processing the pri-shRNA or (ii) the pri-shRNA contains a nuclear import domain that assists in localizing it from the cytoplasm into the nucleus. The recent studies have shown that several cytoplasmic RNA viruses [11,13] are capable of inducing Drosha translocation to the host cytoplasm upon infection and aiding in small RNA (pri-miRNA) processing. However, many other RNA viruses have not been tested for their ability to induce Drosha translocation into the cytoplasm, and there is a possibility of failure of Drosha translocation [14].

Newcastle disease virus (NDV), a member of the family *Paramyxoviridae*, is a species of avian *Orthoavulavirus* with a negative-sense, ss, monopartite RNA genome [15]. NDV infection has been observed across a wide range of bird species with varying susceptibility levels [16]. Due to its potential for oncolysis as well as its economic importance, NDV has recently become a prominent subject of research. Both preclinical and clinical research have extensively utilized NDV as an oncolytic agent [17]. Beyond its direct oncolytic effects, NDV can also be used as a vector for cancer gene therapy. Consequently, various recombinant oncolytic NDVs containing additional transgenes have been developed via reverse genetics. Despite an impressive number of preclinical and clinical studies on the application of NDV in cancer treatment, no study has been reported on the use of this virus as a vector to deliver shRNA, which could potentially further enhance its therapeutic potential, especially in cancer treatment, by silencing the oncogene. Similarly, the replication of NDV remains in the cytoplasm, like most of the RNA viruses. Therefore, we aimed to develop a novel approach to translocate the small RNA (either miRNA or shRNA) produced in the cytoplasm into the nucleus, independent of Drosha. Here, we constructed a recombinant NDV harbouring shRNA with an AU-rich domain at the 3′ end, targeting the mCherry gene (shmCherry) that is constitutively expressed in a previously developed cell line (SW620-mC cell), as a proof of concept [18].

## Theory and implementation

**Fig. 1.**
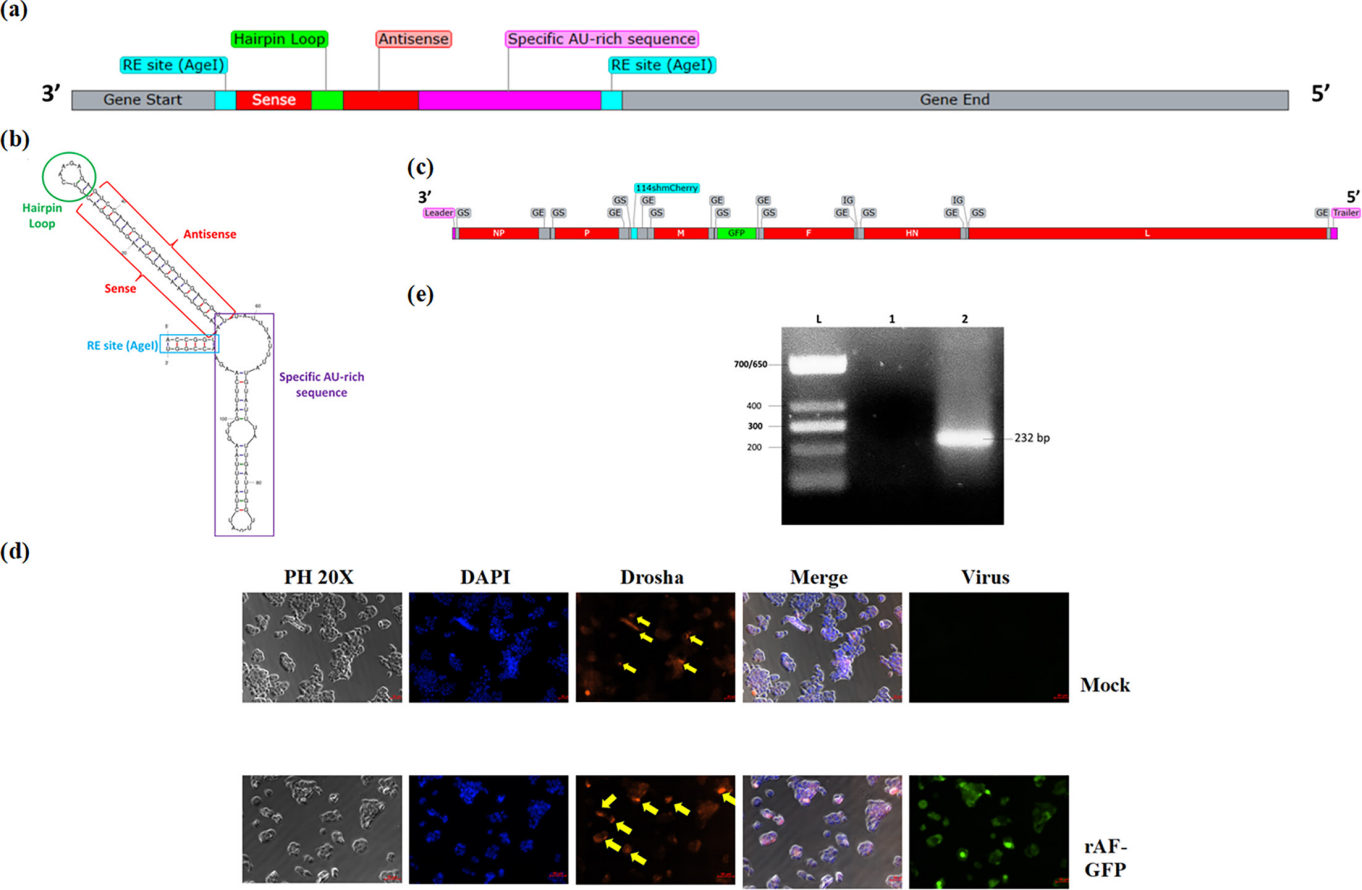
Construction of recombinant NDV (rAF-114shmCherry) and Drosha translocation determination upon NDV infection. (**a**) The complete shRNA sequence that targets mCherry consists of GS and GE of NDV, restriction enzyme sites (*AgeI*), the sense and antisense regions connected by a hairpin loop and a specific AU-rich domain at the 3′ end. The sequence was then verified using blast (https://blast.ncbi.nlm.nih.gov/Blast.cgi) for the recommended algorithms, G+C content (36–52 mol%), sense and antisense specificity [34]. The shRNA design follows the ‘rule of six’ of the *Paramyxoviridae* family, which meant that the number of nucleotides should be divisible by six to allow effective replication [35]. (**b**) Secondary structure of 114shmCherry predicted by mFold. 114shmCherry is able to form a hairpin structure after folding. The AU-rich region at its 3′ end can also form several loops after folding. (**c**) The genome of recombinant NDV, rAF-114shmCherry. (**d**) Drosha localization upon NDV infection. SW620-mC cells were treated without or with NDV (Mock and rAF-GFP), and immunofluorescent staining was performed after 24 h. Green fluorescence indicated the successful infection of the virus. The localization of Drosha (red) was determined by merging with the image of the nucleus, which was stained blue by DAPI, and images were taken using phase contrast at 20X. Scale bar: 50 *µ*m. In the merged image of viral-infected cells, the red and blue colours overlapped with each other, producing a purple colour. This showed that Drosha remained in the nucleus. (**e**) Verification of ZC3H12D gene expression in the cell. RT-PCR on total RNA extracted from SW620-mC was carried out using ZC3H12D-specific primers to further confirm its expression in the cell used in the study. Agarose gel electrophoresis was run to visualize the presence of a band with the expected size of 232 bp. (L) TriDye ultraLow DNA ladder (NEB), (1) negative control (non-template control) and (2) RNA sample extracted from SW620-mC cell.

NDV has been widely used as a viral vector for gene therapy, delivering various types of transgenes to achieve therapeutic purposes. However, there is no study utilizing NDV as viral vectors for shRNA delivery. This is the first study exploring the feasibility of NDV as shRNA viral vectors. The biggest challenge of NDV as a viral vector for shRNA delivery is that the transcription and replication occur solely in the cytoplasm, whereas the microprocessor (Drosha) for the processing of immature small RNA such as pri-miRNA/shRNA is only present in the nucleus. Therefore, to overcome this problem, we designed an shRNA with an AU-rich element (ARE) at its 3′ end (Fig. 1a). It has been shown that the cellular ZC3H12D (Zinc Finger CCCH-Type Containing 12D) protein bound to a specific AU-rich domain at the 3′ end of an RNA was able to facilitate the translocation of the RNA into the nucleus [19]. The mechanism of this novel approach is illustrated in Fig. S1, available in the online Supplementary Material. We hypothesized that an shRNA containing the specific AU-rich domain would attract the binding of the ZC3H12D protein, which would enable translocation into the nucleus for processing by the microprocessor complex to exert its RNAi mechanism. As proof of concept, an shRNA with ARE at its 3′ end was designed to target the mCherry gene (shmCherry) in this study, causing gene silencing via mRNA degradation.

To check whether the designed shmCherry with an AU-rich region can fold into a proper secondary structure, the whole length of the sequence (5′-ACCGGTAACGTCAACATCAAGTTGGACTTCAAGAGAGTCCAACTTGATGTTGACGTTTATTTATTTATGTATTTATTGATTGGTTGATCTATTTAAGTTGATTCAAGAACCGGT-3′) was put into mFold [20]. As shown in Fig. 1(b), the 114shmCherry was able to fold into a hairpin structure with several loops formed by AU-rich regions at its 3′ end. The secondary structure formed by the AU-rich region may play an important role in recognition by the RNA binding protein to aid in translocation into the nucleus. Similar to many essential processes in the cells, such as splicing, transcription, localization and translation, which are affected by the RNA secondary structure [21]. A complete shRNA expression cassette was constructed when shmCherry with ARE was cloned into pUC57-PasI-P-M-PasI cloning vector via AgeI, followed by subcloning into the antigenomic plasmid of NDV, ‘pOLTV5 (rAF-GFP)’, via PasI cloning site to produce pOLTV5 (rAF-114shmCherry). The original wild-type NDV backbone belongs to the strain AF2240, which is velogenic, but the recombinant rAF-GFP has been determined to be mesogenic [22]. This expression cassette contains a gene start (GS) and gene end (GE) at the beginning and end of shRNA, respectively. They act as conserved transcriptional control sequences [23]. The shRNA expression cassette was inserted into the region between the P and M genes of NDV (Fig. 1c), as this is the optimal insertion site in NDV for transgene expression [24]. The rescue of recombinant NDV was performed using reverse genetics as described in [25]. Once verified with a haemagglutination assay, the virus in the allantoic fluid was purified in a sucrose gradient as described in [26], followed by quantification of the viral titre as described in [25].

Previous studies reported that Sindbis virus [11,12], vesicular stomatitis virus, influenza virus [12,27] and canonical viral pathogen-associated molecular pattern (PAMP), dsRNA [12], were able to cause Drosha translocation upon infection or treatment. This Drosha translocation has been linked to the successful production of functional miRNA that leads to targeted gene silencing. Therefore, the ability of NDV to induce Drosha translocation upon infection in SW620-mC cells was investigated using immunofluorescent staining as described in [14]. In our case, the NDV infection on SW620-mC did not trigger Drosha to be translocated out into the host cytoplasm, as Drosha still remained in the nucleus (Fig. 1f). The exact mechanism of Drosha translocation is still unknown. NDV is not the only virus reported with the failure of Drosha translocation. Cytoplasmic localization of Drosha failed to occur when HeLa cells were infected by measles virus [14]. Since the occurrence of Drosha translocation may be due to the production of dsRNA (a viral PAMP), the C protein of the measles virus that prevents dsRNA from being produced in order to evade the antiviral immune response may result in the failure of Drosha translocation.

**Fig. 2.**
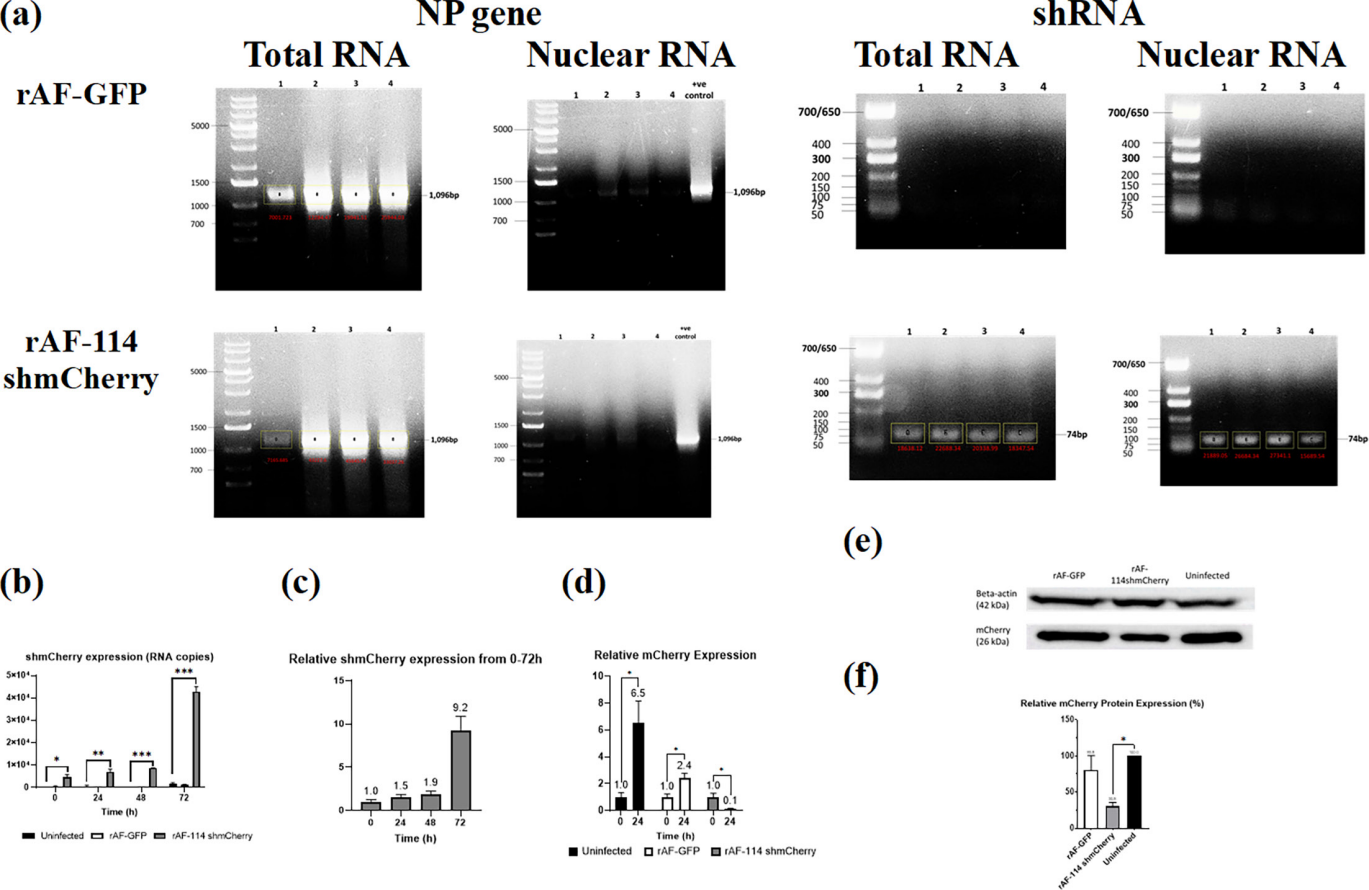
shmCherry localization, expression and functionality in target gene silencing. (**a**) The total RNA and nuclear RNA of uninfected/infected SW620-mC cells were extracted, followed by RT-PCR using NP and shmCherry-specific primers. The band size and intensity were shown in black and red, respectively. The lane was labelled ‘1, 2, 3, 4’, which indicated the harvest timepoint, ‘0, 24, 48, 72 h’, respectively. For the total RNA, the band of the NP gene was only present in the viral-infected cells, indicating the successful infection and replication of the viruses. The band of shmCherry was seen in the rAF-114shmCherry-infected cells, showing the generation of 114shmCherry over time (0–72 h). Besides, the NP gene of NDV is only present in the cytoplasm and can be used to evaluate the presence of cytoplasmic contamination in the nuclei content. For the nuclear RNA, the absence of a band of the NP gene in the figure indicates no cytoplasmic contamination during nuclei isolation (band shown in the figure was positive control of NP gene). The shmCherry, however, was detected in the rAF-114shmCherry-infected cells, suggesting the successful translocation into the nuclei due to the presence of the introduced AU-rich region in its 3′ end. (**b**) Absolute quantification of shmCherry in cells infected with rAF-GFP or rAF-114shmCherry was carried out by RT-qPCR. Viral infection was done at m.o.i. 1 and harvested upon reaching time points (0, 24, 48 and 72 h). RT-qPCR was carried out in technical replicates. The copy number was calculated based on the formula generated from the qPCR standard curve of the shRNA: *X*=(*y*−43.311)/–3.2364, where *X* is the log (shRNA copy number); *y* is the value mean Cq; 43.311 is the *y*-intercept value; −3.2364 is the slope of the standard curve. Data are presented as mean±sem from duplicate determinations. Statistically significant differences between the means were determined by one-way ANOVA followed by Dunnett multiple comparison tests to uninfected cells. Differences were considered significant when *P≤0.05. (**c**) Relative expression of shmCherry in cells infected with rAF-114shmCherry from 0 h to 72 h. Results of qPCR analysis are depicted as shmCherry expression at a particular timepoint relative to shmCherry expression at 0 h in infected cells. The expression of shmCherry produced by rAF-114shmCherry increased from 0 h to 72 h, indicating that shmCherry was continuously produced by the virus over time. (**d**) Relative expression of mCherry from 0 h to 24 h. Results of RT-qPCR analysis are depicted as mCherry expression at a particular timepoint relative to expression at 0 h in the respective group. Data are presented as mean±sem from duplicate determinations. Statistically significant differences between the means were determined by one-way ANOVA followed by Dunnett multiple comparison tests to uninfected cells. Differences were considered significant when *P≤0.05. At 24 h, rAF-114shmCherry downregulated the mCherry expression for around 90%, indicating that shmCherry produced by the virus was functional to cause gene silencing. (**e**) Western blot analysis on mCherry protein in SW620-mC. Viral infection was done at m.o.i. 1, and cell lysate was prepared at 48 h post-infection. Beta-actin was used as the housekeeping protein in this experiment. Upon the infection by rAF-114shmCherry, the mCherry protein in SW620-mC cells was successfully downregulated compared to the uninfected and rAF-GFP-infected cells. (**f**) Relative mCherry protein expression (%). The mCherry protein levels were normalized to beta-actin (housekeeping protein), and the relative mCherry expression in each sample was calculated compared to the uninfected sample. The results are presented as the mean±sem (*n*=3). Statistically significant differences between the means were determined by one-way ANOVA followed by Dunnett multiple comparison tests to uninfected cells. Differences were considered significant when *P≤0.05. rAF-114shmCherry caused significant mCherry protein downregulation (≈70%).

Therefore, instead of relying on the Drosha translocation for shRNA processing, we included an AU-rich region at the 3′ end of shmCherry to enable its translocation by ZC3H12D into the nucleus. The presence of ZC3H12D in SW620-mC was confirmed through the bioinformatic database (https://www.genecards.org/), as shown in GTEx and SAGE (serial analysis of gene expression). A comparable expression of the ZC3H12D gene in colon normal cells was presented in GTEx and SAGE. Then, the ability of nuclear localization of this shRNA with ARE was investigated. The total RNA and nuclear RNA were extracted from the uninfected and viral-infected cells using TRIzol^®^ reagent (Thermo Fisher Scientific, USA) after specific timepoints of virus infection (0, 24, 48 and 72 hpi), followed by reverse transcription PCR (RT-PCR) using different sets of primers targeting the NP gene of NDV and 114shmCherry. The nuclei isolation was performed according to [28] prior to subjection to RNA extraction. From Fig. 2(a), NP bands were only observed in the NDV-infected cells, whereas the shRNA bands were only detected in the rAF-114shmCherry-infected cells. In contrast to the total RNA, there was no band corresponding to the NP gene seen in the nuclear RNA of NDV-infected cells, as depicted in Fig. 2(a). The results also confirmed that there was no cytoplasmic RNA contamination during the nuclear RNA isolation. The amplicon of 114shmCherry was only detected in the rAF-114shmCherry-infected cells, indicating a successful translocation of 114shmCherry into the nucleus. ZC3H12D with different isoforms can be found in both cytoplasm and nucleus, as shown in UniProtKB/Swiss-Prot (**A2A288-ZC12D_HUMAN**). Besides, the translocation of shmCherry by ZC3H12D seemed to be efficient, as the shmCherry was detected in the nucleus at 0 h (1 h post-infection). This suggests that the translocation of shRNA to the nucleus is unlikely to become a limiting factor for mature and functional shRNA production.

Moreover, total RNA of uninfected and infected cells was harvested at indicated timepoints (0, 24, 48 and 72 hpi), and 114shmCherry expression was evaluated using RT-qPCR. In Fig. 2(b, c), the 114shmCherry expression increased significantly over time (0–72 h) as NDV replicated rapidly and produced more 114shmCherry. The gene-silencing effect of shRNA is dose-dependent and has a higher efficiency compared to siRNA [29]. High shRNA expression can ensure effective gene silencing.

Finally, target gene silencing by shRNA was evaluated using RT-qPCR and Western blot analysis to determine mCherry mRNA and protein levels in NDV-infected cells. All primer and probe sequences are summarized in Table S1. The relative mCherry expression of each group was calculated by comparing to its respective baseline value at 0 h. In Fig. 2(d), the highest mCherry expression at 24 h was seen in the uninfected cells as the cells were growing and expressing the gene continuously. In the rAF-GFP-infected cells, there was a lower mCherry expression compared to uninfected cells, as NDV infection can suppress transcription and protein synthesis of host cells. This suppression may be caused by the nuclear localization of the M protein of NDV, which functions similarly as in VSV and human respiratory syncytial virus [30]. Interestingly, rAF-114shmCherry was able to downregulate the mCherry expression at the mRNA level for almost 90% at 24 h, compared to the uninfected and rAF-GFP-infected cells. This indicates that functional shmCherry was successfully produced after translocation into the nucleus for Drosha processing and finally exerted its silencing effect on the target gene, mCherry, via mRNA degradation. The 24 h timepoint was chosen, as the results beyond 24 h may not be accurate, as NDV can cause oncolysis and induce apoptosis in the cancer cell lines, leading to massive cell death [26,31,33]. Besides, to evaluate whether the downregulation of the mCherry gene is reflected at the protein level, a Western blot analysis was performed. Briefly, 20 µg of total cell lysate from each sample (uninfected, rAF-GFP-infected and rAF-114shmCherry-infected) was electrophoresed on 12% SDS-PAGE and transferred onto a PVDF membrane. The proteins were detected using primary antibodies against mCherry (1:5,000; GTX636956) and *β*-actin (1:10,000; GTX109639), respectively, and subsequently with peroxidase-conjugated anti-rabbit secondary antibody (1:10,000; GTX213110-01). Protein detection was performed using WesternBright ECL HRP substrate and visualized using Azure 600 (Azure Biosystems, USA). As shown in Figs 2(e) and S2, Western blot analysis confirmed a similar trend of downregulation of mCherry protein, demonstrating a successful suppression of mCherry expression in SW620-mC cells by rAF-114shmCherry infection, with a reduction of ~70% (Fig. 2f and Table S2). By adding an ARE at the 3′ end of shRNA, the feasibility of NDV as a viral vector for shRNA delivery has been proven, without depending on Drosha translocation for shRNA processing. The silencing efficiency of the NDV vector was compared to the most commonly used DNA viral vector, as shown in Table 1.

**Table 1.** Comparison of silencing efficiency among DNA viral vectors

Viral vector	Target gene	Silencing efficiency at mRNA level (%)	Reference
**Our case (NDV**)	mCherry	≈90	–
**Lentivirus**	Beta-catenin	73	[36]
	DNA polymerase gene of Orf virus	99	[37]
	Survivin	50–80	[38]
	ADAM17	≈50	[39]
	VEGF	53.7	[40]
**Adenovirus**	KCNQ2	80	[41]
	GPR87	≥80	[42]
**AAV**	PSMA2	80	[43]
	EGFR	61–69	[44]

## Conclusion

In conclusion, we have demonstrated that shRNA containing an AU-rich region at its 3′ end, delivered through a novel NDV vector, can successfully translocate into the nucleus, undergo the canonical pathway of small RNA biogenesis and exert its silencing effect on the target gene. It is suggested that the nuclear translocation is dependent on ZC3H12D, rather than Drosha. The silencing efficiency of rAF-114shmCherry on the mCherry gene and protein in SW620f-mC was nearly 90 and 70%, respectively, comparable to the other commonly used DNA viral vectors and RNA viral vectors capable of inducing Drosha translocation. Unlike previously reported RNA viruses, NDV infection in SW620-mC cells did not induce Drosha translocation. This novel approach for translocating shRNA into the nucleus could potentially be applied to other cytoplasmic RNA viruses, allowing for the processing of small RNA (pri-miRNA/shRNA) without relying on Drosha translocation.

## Supplementary material

10.1099/jgv.0.002127 Uncited Supplementary Material 1.

## Abbreviations

AAV: adeno-associated virus
ARE: AU-rich element
GE: gene end
GS: gene start
miRNA: microRNA
NDV: Newcastle disease virus
PAMP: Pathogen-Associated Molecular Pattern
RNAi: RNA interference
RT-PCR: reverse transcription PCR
shRNA: short hairpin RNA
siRNA: small interfering RNA
