# Supplementary material for: Translocating shRNA: a novel approach to RNA interference with Newcastle disease virus as viral vector
Source: J Gen Virol. 2025 Jul 11;106(7):002127. doi: 10.1099/jgv.0.002127 (PMC12248247; doi:10.1099/jgv.0.002127)
Supplement: Uncited Supplementary Material 1. [file jgv-106-02127-s001.pdf]

Supplementary material

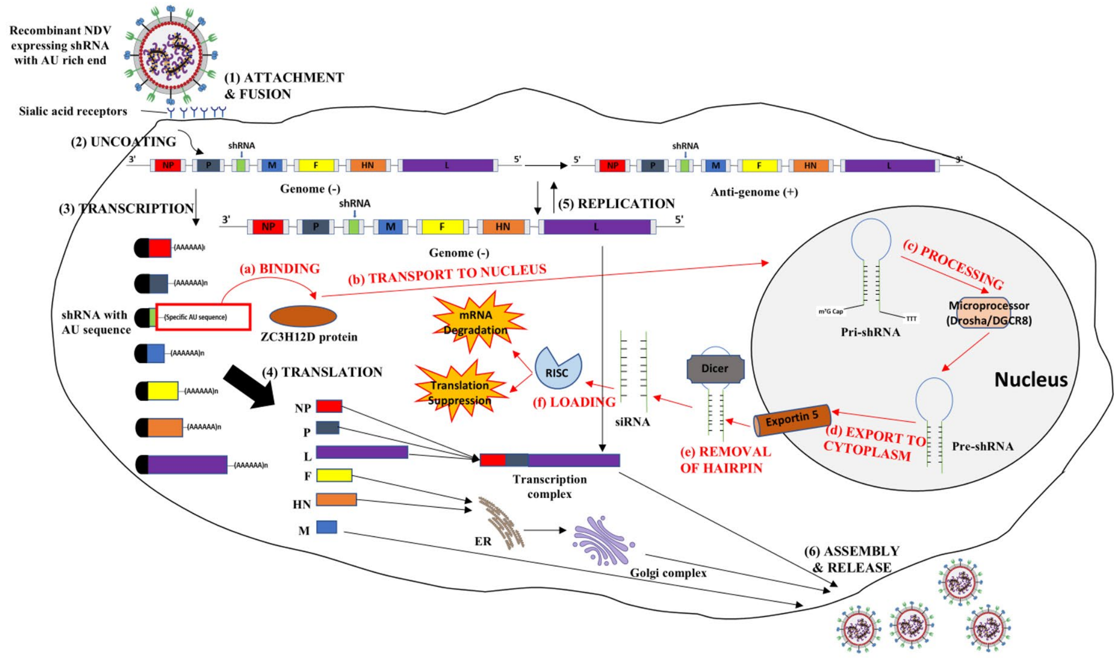

Fig. S1.

Hypothetical mechanism of translocation of shRNA with specific AU-rich region by ZC3H12D protein (described in red alphabets) along NDV replication (described in black numbers). (1) Upon infection, recombinant NDV expressing shRNA with AU-rich region attaches to sialic receptors on the host cell surface, leading to the fusion of viral and cellular membranes. (2) Then the viral nucleocapsid is liberated into the host cell cytoplasm, initiating transcription and replication processes. (3) Transcription is facilitated by the ribonucleoprotein (RNP) complex, comprising the NP, P, and L proteins of NDV, which synthesises viral mRNAs, including the transcript encoding the AU-rich region-containing shRNA. (4) While the produced shRNA continues its process at step (a), the viral mRNAs serve as templates for translation, leading to the production of structural and non-structural proteins such as NP, P, L, F, HN, and M. (5) The newly synthesised RNP complex also plays a pivotal role in viral genome replication, ensuring the production of full-length antigenomic and genomic RNA strands. (6) After undergoing necessary post-translational modifications, particularly of the HN and F proteins, the newly formed viral progeny is assembled and subsequently released from the host cell to propagate infection. (a) Once the shRNA is transcribed, it attracts the binding of ZC3H12D protein due to the presence of AU-rich region at its 3’ end. (b) ZC3H12D protein carries and transports this shRNA to the nucleus for processing. (c) The primary transcript of shRNA (pri-shRNA) is cleaved and processed by microprocessor (Drosha/ DGCR8) in the nucleus, producing precursor shRNA (pre-shRNA). (d) Pre-shRNA is then exported to cytoplasm via Exportin 5. (e) siRNA that is complementary to the target gene is produced upon the removal of hairpin in shRNA by Dicer. (f) Finally, the guide strand of siRNA is loaded onto RISC and causes gene silencing via either mRNA degradation or translation inhibition, depending on the degree of complementary [1].

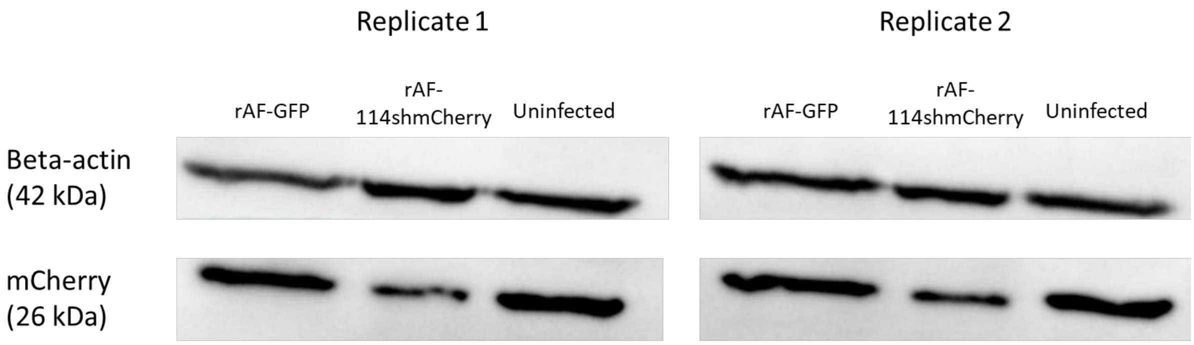

Fig. S2.

Western blot analysis on mCherry protein in SW620-mC. Viral infection was done at MOI 1 and cell lysate was prepared at 48 h post-infection. Beta-actin was used as the housekeeping protein in this experiment. Upon the infection by rAF-114shmCherry, the mCherry protein in SW620-mC cells was downregulated.

Table S1.

Primer sequences used in RT-PCR and RT-qPCR

Target gene	Forward primer sequence (5’-3’)	Reverse primer sequence (5’-3’)
shmCherry	ACGGGTAGAAGAGTTCGGA	TCTTGAAGTCCAACTTGATGTTGAC GTTAC
NP	ATGAGGCTACACTGAC	TCTCGCATGCTGTTCG
ZC3H12D	CTTCTCTTGCCGGGGAATCA	CTTCACGATGTAGCGGTCGT
shmCherry qPCR	ACGGGTAGAAGAGTTCGGAT	TCTCTTGAAGTCCAACTTGATGT
shmCherry qPCR probe	6FAM-TTGGCACATATCCGGACCGGTAAC-3'Zen/Iowa Black™ FQ	
mCherry qPCR	TTGGACATCACCTCCCACAAC	CCTCGGCGCGTTCGTA
mCherry qPCR probe	6FAM-ACTACACCATCGTGGAAC-MGB/NFQ	

Table S2

Relative mCherry protein expression of each replicate in SW620 (%)

Sample	Relative mCherry Protein Expression (%)			
	**1**	**2**	**3**	**Average**
**rAF-GFP**	57.94	92.28	92.24	80.82
**rAF-114shmCherry**	28.08	27.82	36.60	30.83
**Uninfected**	100.00	100.00	100.00	100.00

Supplemental References

1. **Torrecilla J, Rodríguez-Gascón A, Solinís MÁ, del Pozo-Rodríguez A**. Lipid nanoparticles as carriers for RNAi against viral infections: current status and future perspectives. *Biomed Res Int* 2014;2014:161794.
